# Posterior stability of the shoulder depends on acromial anatomy: a biomechanical study of 3D surface models

**DOI:** 10.1186/s40634-023-00623-x

**Published:** 2023-06-01

**Authors:** Bettina Hochreiter, Silvan Beeler, Simon Hofstede, Bastian Sigrist, Jess G. Snedeker, Christian Gerber

**Affiliations:** 1 Department of Orthopaedics, University of Zurich, Balgrist University Hospital, Zurich, Switzerland; 2 Department of Orthopaedics, Biomechanical Research Laboratory, University of Zurich, Balgrist Campus, Zurich, Switzerland; 3 Research in Orthopaedic Computer Science (ROCS), University of Zurich, Balgrist University Hospital, Zurich, Switzerland; 4 Balgrist Campus, Orthopaedic Research Center, Zurich, Switzerland

**Keywords:** Posterior shoulder instability, Humeral head subluxation, Static posterior, Walch B1, Acromion, Shoulder, Anatomy

## Abstract

**Purpose:**

Primary glenohumeral osteoarthritis is commonly associated with static posterior subluxation of the humeral head. Scapulae with static/dynamic posterior instability feature a superiorly and horizontally oriented acromion. We investigated whether the acromion acts as a restraint to posterior humeral translation.

**Methods:**

Five three-dimensional (3D) printed scapula models were biomechanically tested. A statistical shape mean model (SSMM) of the normal scapula of 40 asymptomatic shoulders was fabricated. Next, a SSMM of scapular anatomy associated with posterior subluxation was generated using data of 20 scapulae (“B1”). This model was then used to generate three models of surgical correction: glenoid version, acromial orientation, and acromial *and* glenoid orientation. With the joint axially loaded (100N) and the humerus stabilized, an anterior translation force was applied to the scapula in 35°, 60° and 75° of glenohumeral flexion. Translation (mm) was measured.

**Results:**

In the normal scapula, the humerus translates significantly less to contact with the acromion compared to all other configurations (*p* < .000 for all comparisons; i.e. 35°: “normal” 8,1 mm (± 0,0) versus “B1” 11,9 mm (± 0,0) versus “B1 Acromion Correction” 12,2 mm (± 0,2) versus “B1 Glenoid Correction” 13,3 mm (± 0,1)). Restoration of normal translation was only achieved with correction of glenoid *and* acromial anatomy (i.e. 75°: “normal” 11 mm (± 0,8) versus “B1 Acromion Correction” 17,5 mm (± 0,1) versus “B1 Glenoid Correction” 19,7 mm (± 1,3) versus “B1 Glenoid + Acromion Correction” 11,5 mm (± 1,1)).

**Conclusions:**

Persistence or recurrence of static/dynamic posterior instability after correction of glenoid version alone may be related to incomplete restoration of the intrinsic stability that is conferred by a normal acromial anatomy.

**Level of Evidence V:**

biomechanical study

**Supplementary Information:**

The online version contains supplementary material available at 10.1186/s40634-023-00623-x.

## Background

The reasons for static posterior subluxation of the humeral head – defined as posterior glenoid wear with a glenohumeral subluxation index of > 55% as measured on CT scans [11] – associated with B and C type glenoids [18, 20] and eccentric osteoarthritis (OA) are not well understood. A multifactorial etiology is postulated [6, 15, 18, 20]. Osseous changes (glenoid version, anteriorly displaced glenoid vault, acromial morphology), soft tissue factors (rotator cuff muscle imbalance, anterior capsular stiffness) and combinations thereof are held responsible [5]. Currently, correction of static posterior subluxation is attempted by restoring normal glenoid version using scapular neck opening wedge osteotomies. However, this neither consistently corrects posterior subluxation nor does it prevent progression of OA[4, 16]. It is therefore urgent to identify a treatment concept with the potential to durably restore joint concentricity and to decelerate or arrest progressive eccentric OA.

In addition to typically increased glenoid retroversion, scapular anatomy of shoulders with posterior instability and type B glenoids is characterized by abnormal acromial anatomy: Meyer [14, 15] and Beeler [3] documented substantial and consistent differences between the acromion of normal shoulders and shoulders with static posterior subluxation, as well as between stable shoulders and shoulders with dynamic posterior instability. Analysis of posteriorly unstable and statically subluxated shoulders revealed them to be significantly different from normal shoulders in terms of glenoid version, glenoid inclination, posterolateral acromial shape, position and orientation of the acromion. The acromion in these pathologic shoulders was superiorly and more horizontally oriented [2, 14, 15], implying a reduced posterior "coverage" of the humerus. Rationally, reduced posterior coverage could potentially decrease resistance to translation in response to a posterior load on the humerus.

It was therefore the purpose of this study to experimentally test the plausibility of the concept that the acromion acts a restraint to posterior humeral translation and whether the efficacy of this restraint may differ in anatomical variants that typify static or dynamic posterior instability. To answer these questions, the following hypotheses were tested:

1. Posterior acromial morphology significantly affects posterior humeral head translation
2. Less posterior acromial coverage of the humeral head contributes more to the force–displacement behavior of the shoulder than glenoid retroversion
3. Correction of glenoid retroversion alone does not restore normal force–displacement behavior of a typical, posteriorly unstable shoulder
4. Correction of acromial and glenoid (version and inclination) orientation to normal restores force–displacement behavior

## Methods

Approval for the study was obtained from the ethical committee responsible for our institution in Zurich (Basec No. KEK-ZH-Nr.2020–01558).

### Study design

Bioinspired phantoms of both stable shoulder anatomies and those predisposed to instability were created from data of a previous study [3]. Here, a statistical shape mean model (SSMM) of a “normal” scapula was created by synthesizing CT data of 40 asymptomatic shoulders. These 40 patients (20 women, 20 men; age 45–65 years) had CT scans in the course of polytrauma treatment without affection of the upper extremities. CT scans with visible bony defects of the scapula/humerus, osteoarthritis, rotator cuff tears, glenoid dysplasia (Walch type C glenoid [19] and glenoid dysplasia according to Weishaupt [21]) and a history of any shoulder pathology in the past were excluded.

**Fig. 1:**
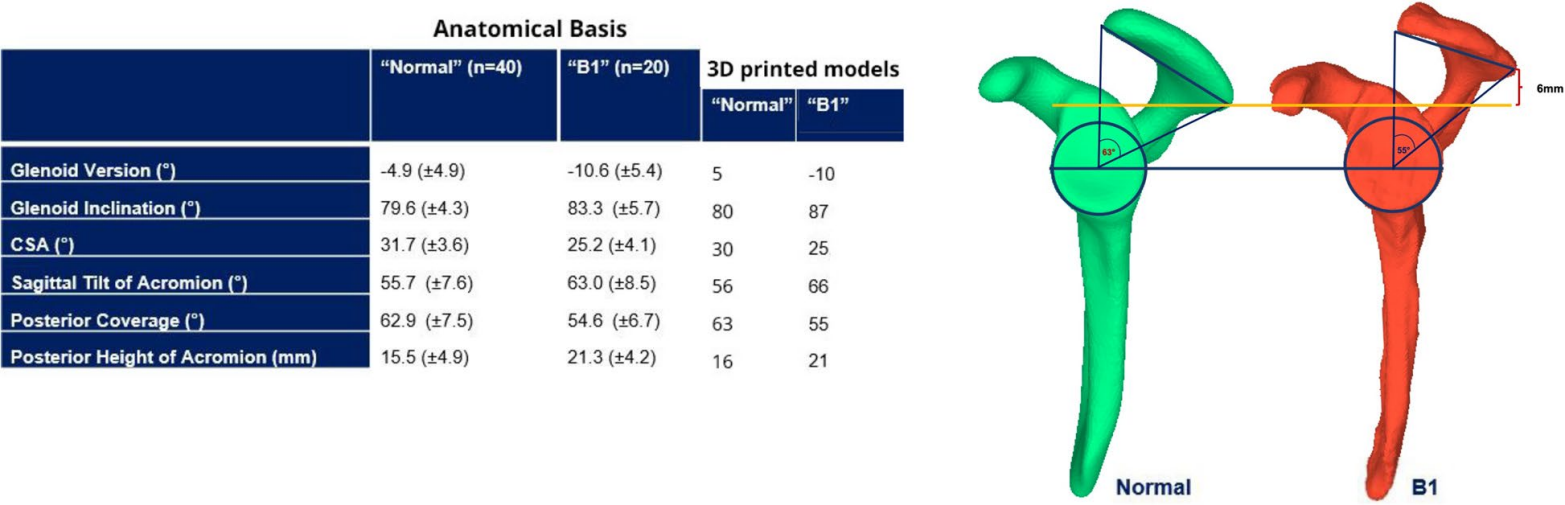
The first two rows show anthropometric data (mean values and SD) for “normal” scapulae (= statistical shape mean model; from Beeler [3]) and “B1” scapulae (according to Walch [17]) with static posterior subluxation (from Beeler [3]). The last two rows show the data of the two printed and tested models. Note the differences in posterior height of acromion and posterior coverage (6 mm higher acromion – measured as the distance between the posterolateral acromial edge and a line through the center of the glenoid drawn perpendicular to the scapular plane; and 8° less coverage – measured as an angle between a line drawn parallel to the scapular plane and a line to the posterolateral acromial edge) between a “normal” scapula and a “B1” scapula

The SSMM was generated with a commercial software (Shapemeans, Allschwil, Switzerland) and the 3-D mean model was obtained. This mean model defined normal anatomy (Fig. 1).

The same procedure was followed for 20 statically, posteriorly subluxated, pre-arthritic shoulders (classified as B1) [18]. Static posterior humeral head subluxation was defined as a glenohumeral subluxation index of > 55% as defined by Jacxsens et al. [11] on a CT scan taken with the patient supine at midglenoid level. However, the CT scans of these shoulders did not always image the entire scapula. The CT scans allowed to determine all key anatomical parameters (Fig. 1) but not to create an SSMM of the entire scapula. Therefore, the clinical example of a B1 shoulder with the best fit of all mean values of this group and a completely imaged scapula was selected to represent the statically, posteriorly subluxated shoulder (“model B1”) (Fig. 1).

**Fig. 2:**
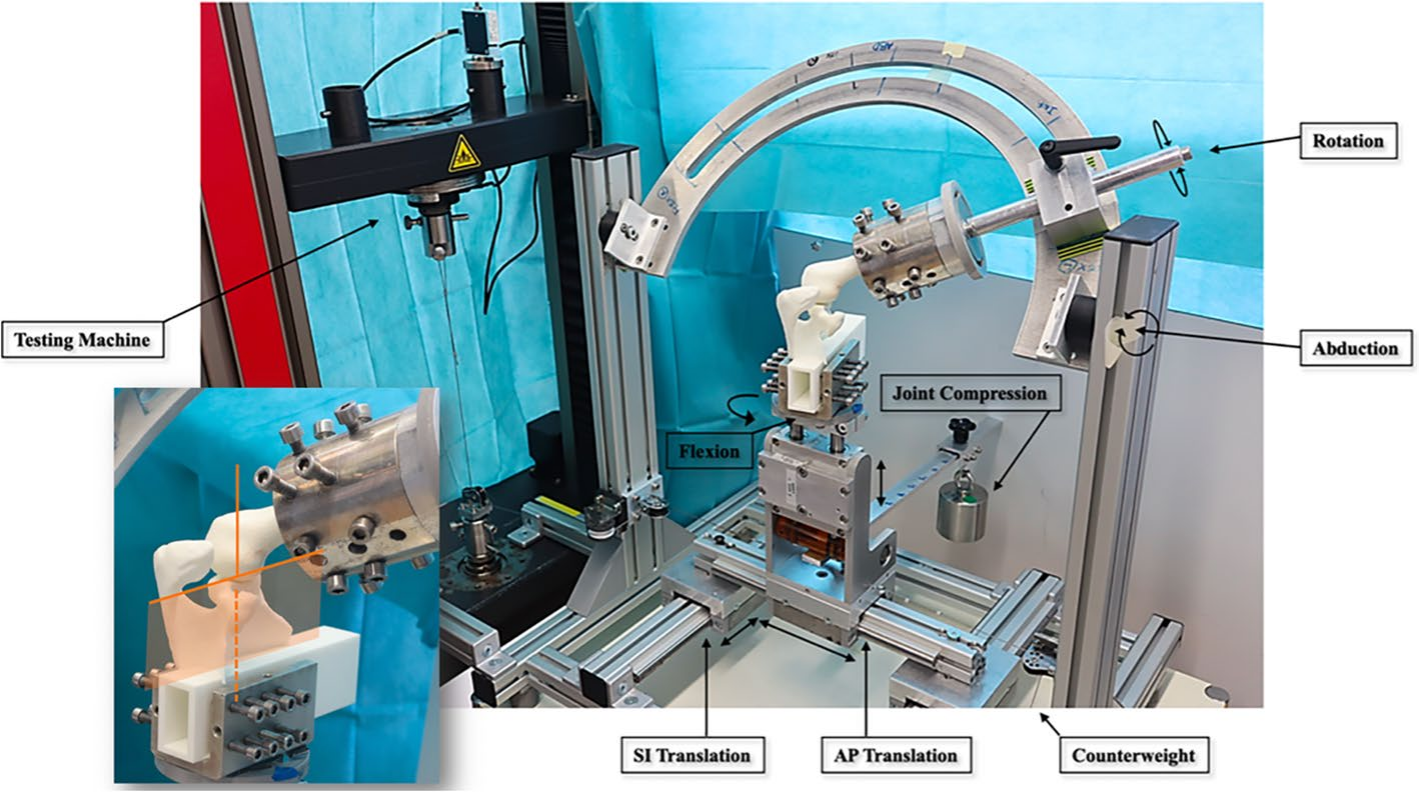
A box aligned in the scapular plane (165 × 50x30mm), was placed around the virtual scapular blade (with the planning software CASPA). Differences in glenoid version and inclination could thereby be reproduced with reference to the scapular or horizontal plane, respectively. This was done in order to prevent incorrect positioning when mounting the models on the biomechanical apparatus. The scapula was fixed in a vice at the box level, with the scapular plane (orange plane) oriented strictly vertically, the glenoid facing upward. This defined glenoid version. Glenoid inclination was incorporated into the printed models with respect to the horizontal plane according to the predetermined values for all models. The scapular models were fixed on a plate on top of a bearing plate which could be levered upward to exert a compression force of 100N. With the humerus fixed, the anteroposterior (AP) and superoinferior (SI) translatable plate with its fixed scapula was rotated to simulate different shoulder flexion angles (60°, 90°, 120°)

Data for the “normal” composite model and the “typical B1 model were imported into the MIMICS software (Materialize, Leuven, Belgium) and semi-automatic 3D segmentation was performed (B.S.). The reconstructed scapula models were then imported into the planning software CASPA (Computer Assisted Surgery Planning Application Version 5.0, in-house development Balgrist CARD AG) and oriented in a standardized scapular plane defined by: the center of a best-fit circle of the inferior glenoid, the intersection of the scapular spine with the medial border and the inferior tip of the scapula. To prevent incorrect positioning of the printed models on the biomechanical apparatus, a box (165 × 50x30mm), aligned in the scapular plane, was designed around the virtual scapular blade (Fig. 2). Differences in glenoid version and inclination could thereby be reproduced with reference to the scapular or horizontal plane, respectively. The distance from the center of the glenoid to the box was standardized (105 mm). Before the bioinspired shoulder phantoms were 3D printed they were anatomically scaled to correspond to the size of the "normal" model. The glenoid surface characteristics of the "normal" SSMM was applied to all other models to eliminate glenoid surface properties as a confounding factor. Also, according to the SSMM of the normal scapula, a humeral head with a 44 mm diameter was used [3].

Thus, the following five physical models were printed using 3-D selective laser sintering (EOS Formiga P100, Munich, Germany; polyamide 12 powder with a layer thickness of 0.12 mm):

1. a statistical shape mean model biomechanical phantom representing a *normal scapula* (“normal”: composite anatomy created by averaging)
2. a best fit *B1 scapula model* biomechanical phantom representing static posterior subluxation (“B1”: typical scapula that best fit to anatomical parameters of the class). This base anatomy was modified to include:
  a “B1” after correction of *glenoid version* to “normal”
  b “B1” after correction of the *acromial orientation* to “normal”
  c “B1” after correction of *acromial and glenoid (version and inclination) orientation* to “normal”

### Biomechanical Test – Setup

The biomechanical setup allowed positioning of the joint with six degrees of freedom, as previously described in earlier biomechanical studies on shoulder instability [10] (Fig. 2). Briefly, the scapular phantom was fixed in a vice, with the scapular plane oriented vertically. The scapula was mounted on top of a bearing plate which could be rotated to simulate different flexion angles. The humerus was fixed in a cylinder on an arc on top of the simulator in a neutral humeral rotation [17]. The arc was adjusted to a glenohumeral abduction of 45° (corresponding to the abduction angle during shoulder flexion [13]). Two linear motion slides enabled antero-posterior and inferior translation of the scapula. Superior translation was blocked to prevent the scapula from moving cranially at humero-acromial contact. To translate the scapula, the antero-posterior linear translator was connected to a uniaxial material testing machine (Z010 TH, ZwickRoell, Ulm, Germany) equipped with a 10 kN load cell. The position of the scapula simulated 35°, 60° and 75° glenohumeral flexion (corresponding to 60°, 90°, 120° shoulder flexion [13]). For better comprehensibility, the flexion angles in the manuscript are referred to as shoulder flexion angles (60, 90, 120°) instead of glenohumeral flexion angles (35, 60, 75°). Polyamide 12 powder, also known as nylon, was used for 3D printing of the scapulae. As the surface texture of nylon is somewhat rough a rubber balloon was firmly applied to the articulating surfaces (humeral head, glenoid, acromion) and prior to each measurement the rubber surfaces were lubricated with lubricating oil (MOTOREX, Supergliss K68) to reduce friction. A static compression force of 100N – applied through a lever arm system – centered the humeral head in the glenoid concavity [12]. The antero-posterior alignment was visually verified and the position recorded by the testing machine, ensuring reproducible starting positions between repetitions.

The scapulae were pulled anteriorly, corresponding to a posterior translation of the humeral head, at a rate of 1 mm/s. The endpoints of measurement were either an achieved contact force of 40N between the acromion and humerus or a posterior dislocation. Both, the 40N of contact force and the amount of posterior translation (until 40N or posterior dislocation were noted) were measured by the ZwickRoell testing machine and a force–displacement curve was produced. A registered contact force of 40N led to deformation of the acromion, while posterior dislocation was defined as a sudden drop in the registered force–displacement curve and was controlled visually. After each measurement, the starting point was automatically approached by the machine. Every model was tested 3 times in 60°, 90° and 120° of shoulder flexion. Therefore, there were 9 tests per model and 45 tests in total.

### Biomechanical and statistical analysis

Force—displacement data were recorded with the TestXpert II software (ZwickRoell, Ulm, Germany), and further processed using Microsoft Excel (Professional Plus 2019, Microsoft Corporation, Washington, USA). Displacement (mm) was calculated from the starting point (decentralization) to the end point (acromion contact or dislocation) after loading.

Statistical Analysis was primarily performed in a descriptive fashion. Mean values, standard deviations (SD) and 95% confidence intervals were calculated for every three test runs per flexion angle per model. Furthermore, a one-way ANOVA was conducted to assess the effects of the model on posterior translation until acromion contact or dislocation, within each flexion angle. Post-hoc comparisons were performed for selected pairs, and the respective *p*-values were Bonferroni-corrected. The analysis was performed with SPSS (IBM Corp. Version 27). *P*-values < 0.05 were considered statistically significant.

## Results

**Fig. 3:**
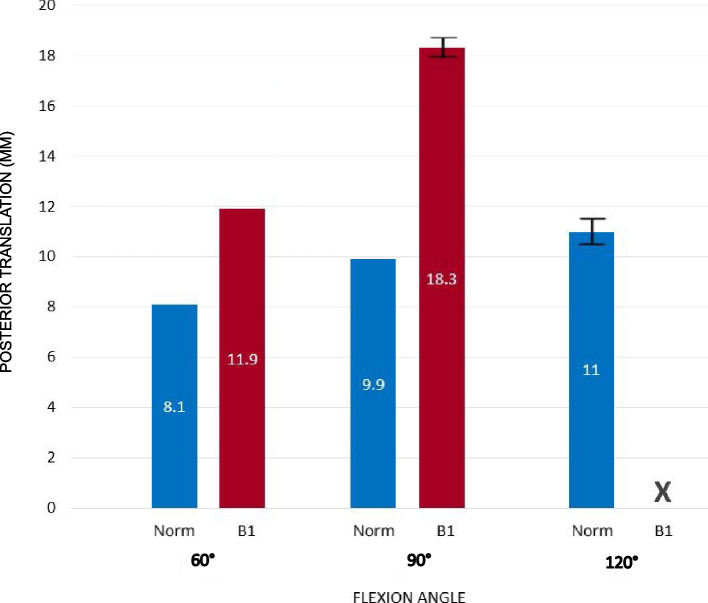
Displacement until acromion contact at all tested flexion angles for normal and pathological scapulae. In 60° and 90° flexion, the normal and the “B1” model had acromion contact. However, the “B1” model showed significantly more translation until acromion contact occurred. In 120° flexion, the “B1” model showed significantly more posterior translation until acromion contact occurred and eventually dislocated while the “normal” model had acromion contact and remained stable. Values in mean, bars show SD

**Fig. 4:**
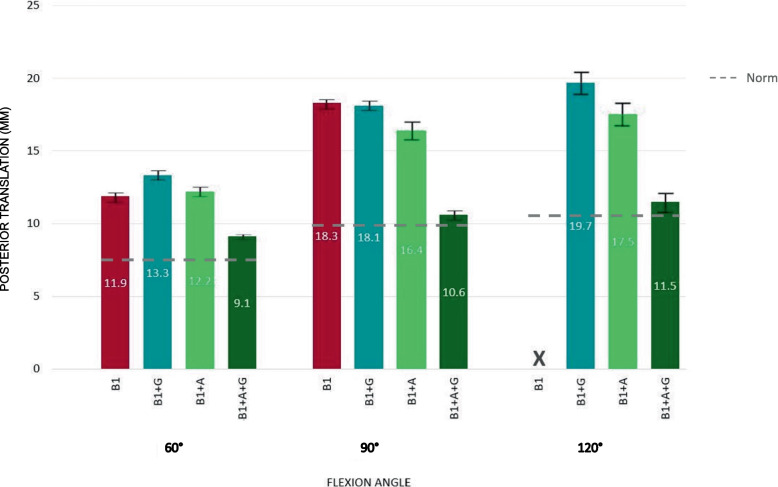
Displacement until acromion contact at all tested flexion angles for “B1” scapula and corrected versions of “B1” scapula. The "B1 Acromion Correction" showed less translation compared to the “B1 Glenoid Correction” model. However, in all tested flexion angles only the combined “B1 Acromion + Glenoid Correction” had comparable posterior translation to the “normal” shoulder model. Values in mean, bars show SD

Simulated shoulder instability increased with increasing higher glenohumeral flexion angles, and was observed to depend on both glenoid and acromion anatomy (Table 1, Fig. 3 and 4). Results from the biomechanical testing on the anatomical phantoms are presented below according to the tested anatomical orientation of the glenohumeral joint.

**Table 1:** Overview of all tested situations (five models at three shoulder flexion angles) and information on whether and after which posterior translation (mm) the humeral head was either in contact with the acromion or dislocated posteriorly

		**Contact with Acromion**			**Posterior Dislocation**		
		occurred	mm^a^	95% CI	occurred	mm^a^	95% CI
**60° Flexion**	**Normal**	yes	8.1 (0.0)	8.1–8.1	no	n.a	n.a
	**B1 Acromion + Glenoid Correction**	yes	9.1 (0.2)	8.8–9.5	no	n.a	n.a
	**B1**	yes	11.9 (0.0)	11.9–11.9	no	n.a	n.a
	**B1 Acromion Correction**	yes	12.2 (0.2)	11.6–12.7	no	n.a	n.a
	**B1 Glenoid Correction**	yes	13.3 (0.1)	13.2–13.5	no	n.a	n.a
**90° Flexion**	**Normal**	yes	9.9 (0.1)	9.8–10.1	no	n.a	n.a
	**B1 Acromion + Glenoid Correction**	yes	10.6 (0.2)	10.1–11.1	no	n.a	n.a
	**B1**	yes	18.3 (0.3)	17.5–19.1	yes	22.3 (0.2)	21.9–22.7
	**B1 Acromion Correction**	yes	16.4 (0.5)	15.2–17.7	yes	28.4 (1.7)	24.1–32.7
	**B1 Glenoid Correction**	yes	18.1 (0.2)	17.5–18.6	yes	27.0 (0.2)	26.6–27.4
**120° Flexion**	**Normal**	yes	11 (0.8)	9.1–12.9	yes	25.2 (0.6)	23.8–26.6
	**B1 Acromion + Glenoid Correction**	yes	11.5 (1.1)	8.8–14.2	yes	25.7 (0.7)	23.9–27.5
	**B1**	no	n.a	n.a	yes	22.3 (0.2)	21.9–22.7
	**B1 Acromion Correction**	yes	17.5 (0.1)	17.4–17.7	yes	26.5 (0.2)	26.1–26.9
	**B1 Glenoid Correction**	yes	19.7 (1.3)	16.5–22.8	yes	26.6 (1.2)	23.6–29.6

^a^Translation until event occurs; values in mean (SD); *CI* confidence intervals; *n.a.* not available

### 60° shoulder flexion

In 60° shoulder flexion, all shoulders were stable with tests terminating after achieving 40N acromiohumeral contact force. The “B1” model showed 4 mm more posterior translation on average at acromion contact than the “normal” model (“normal” 8.1 mm (± 0.0) versus “B1” 11.9 mm (± 0.0)); (Fig. 3, Video 1 vs. Video 3). The "B1" (11.9 mm (± 0.0)), "B1 Glenoid Correction" (13,3 mm (± 0.1)) and the "B1 Acromion Correction" (12,2 mm (± 0.2)) models showed increased translation as well compared to the “B1 Acromion + Glenoid Correction” group (9.1 mm (± 0.2)) (*p* < 0.001 for all comparisons) (Table 1).

### 90° shoulder flexion

In 90° shoulder flexion, three of five models dislocated. Only the "normal" and "B1 Acromion + Glenoid Correction" did not dislocate (Video 2). Acromion contact was achieved in all models. The “B1” (Video 4), "B1 Glenoid Correction" as well as the “B1 Acromion Correction” model showed acromion contact significantly later than the other models (*p* < 0.001 for all comparisons; “normal” 9.9 mm (± 0.1) versus “B1 Glenoid Correction” 18.1 mm (± 0.2) versus “B1 Acromion Correction” 16.4 mm (± 0.5)). The “B1 Acromion Correction” model showed acromion contact after 16.4 mm (± 0,5) mm of posterior translation which is 1.7 mm and 1.9 mm less compared to the “B1” or “B1 Glenoid Correction” models (*p* < 0.05 for both comparisons), respectively. The "B1" model dislocated on average 6 mm earlier than the "B1 Acromion Correction" model (“B1” 22.3 mm (± 0.2) versus “B1 Acromion Correction” 28.4 mm (± 1.7)).

### 120° shoulder flexion

In 120° shoulder flexion all models dislocated. Only the "B1" model had no acromion contact and therefore dislocated significantly earlier compared to all other models (*p* < 0.05 for all comparisons; “normal” 25.2 mm (± 0.6) versus “B1” 22,3 mm (± 0.2) versus “B1 Acromion Correction” 26.5 mm (± 0.2) versus “ B1 Glenoid Correction” 26.6 mm (± 1.2)). The “B1 Acromion Correction” and the “B1 Glenoid Correction” model showed acromion contact significantly later than the other models (*p* < 0.000 for all comparisons; “normal” 11 mm (± 0.8) versus “B1 Acromion Correction” 17,5 mm (± 0.1) versus “B1 Glenoid Correction” 19.7 mm (± 1.3) versus “B1 Glenoid + Acromion Correction” 11.5 mm (± 1.1)).

In all situations tested, acromion correction alone resulted in a shorter translational distance to acromion contact compared to glenoid correction alone (Fig. 4). However, this difference was only significant in 90 and 120° of flexion (*p* < 0.05).

## Discussion

The most important finding of our study is that posterior acromial morphology seems to significantly affect humeral translation in response to antero-posterior loading of the joint. Our biomechanical models represented only osseous structures and tested whether the acromion provides passive mechanical resistance to posterior translation, or even dislocation of the humerus. The most interesting finding was that in 60° of shoulder flexion, the humeral head had contact with the acromion in all models and this contact prevented posterior dislocation. If dislocation did not occur in the B1 scapula, identical antero-posterior loading was associated with substantially increased posterior translation compared to the normal scapula: this suggests that in everyday activities below the horizontal plane, the humeral head can translate about 4 mm more posteriorly on the glenoid once capsular laxity and muscle activity allows to do so. If anterior elevation is increased to 90°, posterior translation in the B1 with respect to the normal shoulder essentially doubles before posterior dislocation occurs. This corresponds to the clinical experience which – with positional posterior subluxation occurring between 90–110° [9] of flexion in combination with the presented data – would be compatible with a hypothesis that posterior soft tissue laxity develops secondarily, due to the decreased resistance to posterior translation. Correction of increased glenoid retroversion alone did neither reduce posterior translation to normal nor prevent posterior dislocation in this study. This may explain why posterior opening wedge osteotomies or posterior J-grafts fail to consistently and durably recenter the humeral head [7, 16].

Posterior acromial morphology appears to contribute more to the force–displacement behavior of the posteriorly unstable shoulder than increased glenoid retroversion. In all situations tested, acromion correction alone resulted in a shorter translational distance to acromion contact compared to glenoid version correction alone.

Correction of increased glenoid retroversion alone seems to neither significantly reduce posterior translation until acromion contact nor prevent posterior dislocation in a B1 scapula. The literature focuses on correction of glenoid version [7, 16] but does not consider glenoid inclination. Type B1 shoulders can, however, not only exhibit increased glenoid retroversion but also increased inferior tilt [3] (Fig. 1). Our "B1 Acromion + Glenoid Correction" model corrected both deformities, did thus not correspond to an isolated correction of glenoid version. With a more superior starting point of humeral-head-glenoid-contact, the humeral head is brought closer to the acromion leading to earlier contact with the acromion.

Correction of acromial morphology and glenoid orientation restores near normal force–displacement behavior in this experimental setup. There were no significant differences between the “normal” model and the “B1 Acromion + Glenoid Correction” model in all tested situations. These results are in favor of a comprehensive correction of extraarticular scapular deformities.

The etiology underlying dynamic and static posterior instability with eccentric OA of the shoulder has been debated and speculated upon for years [5]. Several studies have demonstrated that acromial anatomy typically differs substantially between patients with a healthy shoulder, shoulders with eccentric OA, or posterior instability [1, 2, 14, 15]. The acromion of the patient groups that present with posterior instability is characterized by a high and flat roof with less posterolateral coverage of the humeral head. This study tests the radiological observations of Meyer [14, 15]. and Beeler et al. [1–3] in a biomechanical setting and provides evidence that these observations are indeed consistent with increased posterior translation and posterior instability. The clinical relevance of this study is that it demonstrates that such characteristic anatomical features of the acromion can affect its efficacy as a restraint to posterior translation of the humerus during glenohumeral elevation. Our study results could explain the high failure rates of conventional operative treatment for posterior instability and incipient eccentric OA [4, 7, 16]. They would suggest combined (glenoid and acromion) osteotomies planned on quantitative 3-D analyses of individual B1 scapulae and planned restoration of the anatomy as near as possible to the normal shoulder [8].

### Limitations

The main limitation of the present study is that the shoulder joint was reduced to a model of the osseous joint partners and superior translation was blocked, neglecting potentially relevant contributions of some passive (capsulo-ligamentous) and active (muscular) contributors to shoulder stability. This proof-of-concept study is, in this sense, only a crude representation of functional shoulder anatomy. The model would benefit from refinement in future studies that mimic soft tissue contributions to shoulder stability in patients.

The advent of image processing and 3D printing technology is opening up many opportunities in patient-specific applications in orthopedics such as for the creation of anatomic models for surgical planning and training, education, PSIs, and 3D-printed custom implants. However, 3D printed bioinspired phantoms have not yet been used to answer biomechanical questions. Anterior and posterior glenohumeral instability have been tested in numerous in vitro studies with their respective limitations. Nevertheless, we consider the study design used to be valid, as it allows testing of purely osseous factors. This would not have been possible in a cadaveric model.

## Conclusions

A normal acromial anatomy might act as a restraint to posterior humeral head translation. Persistence or recurrence of static and/or dynamic posterior instability after correction of glenoid version alone may be related to incomplete restoration of the intrinsic stability that is conferred by a normal acromial anatomy.

### Supplementary Information

**Additional file 1: Videos1-4***. *The pressuredistribution on glenoid and acromion surfaces was assessed by separatemeasurements using TekScan 4000 pressure sensors (TekScan Inc., South Boston,Massachussetts, USA). One-point calibration of the sensors with 50 N wasperformed using a custom-built jig mounted on a material testing machine (Z010TH, ZwickRoell, Ulm, Germany). The pressure sensors were placed in astandardized manner on the glenoid in supero-inferior alignment and on theundersurface of the acromion in anteroposterior alignment. The pressure sensorswere placed so that they were flush with the postero-inferior margin on boththe glenoid and acromion, and were secured in place with bi-adhesive tape. Thepressure sensors were used to verify acromion contact and document the path ofthe humeral head on the glenoid as well as the lower surface of the acromion.Data was not evaluated quantitively.Videos 1 and 2 show the “normal” and Videos 3 and 4 show the “B1” modelin 35° and 60° of glenohumeral flexion. Note the superior starting point in the“normal” model, which is attributable to the greater upward tilt of theglenoid. This leads to relatively more posterolateral acromial coverage of thehumeral head. The “B1” model shows significantly more posterior translationuntil acromion contact. The contact area of the humeral head with the acromionis further lateral and inferior compared to the “normal” model. Furthermore,the pathological model dislocates in 60° of glenohumeral flexion whereas the“normal” model does not.

**Additional file 2.**

**Additional file 3.**

**Additional file 4.**

## Data Availability

The datasets used and analysed during the current study are available from the corresponding author on reasonable request.
