# Correction: Posterior stability of the shoulder depends on acromial anatomy: a biomechanical study of 3D surface models

**DOI:** 10.1186/s40634-023-00711-y

**Published:** 2023-12-26

**Authors:** Bettina Hochreiter, Silvan Beeler, Simon Hofstede, Bastian Sigrist, Jess G. Snedeker, Christian Gerber

**Affiliations:** 1 Department of Orthopaedics, University of Zurich, Balgrist University Hospital, Zurich, Switzerland; 2 Department of Orthopaedics, Biomechanical Research Laboratory, University of Zurich, Balgrist Campus, Zurich, Switzerland; 3 Research in Orthopaedic Computer Science (ROCS), University of Zurich, Balgrist University Hospital, Zurich, Switzerland; 4 Balgrist Campus, Orthopaedic Research Center, Zurich, Switzerland

**Correction: J Exp Orthop 10, 59 (2023)**

10.1186/s40634-023-00623-x

Following publication of the original article [1], the authors reported that the cited number of the ethical approval “2020-01558” is incorrect. It should be corrected to “2020-00389”. The original article has been corrected.
